# Mastocytosis patients' cognitive dysfunctions correlate with the presence of spindle‐shaped mast cells in bone marrow

**DOI:** 10.1002/clt2.12093

**Published:** 2022-01-11

**Authors:** Natalia Spolak‐Bobryk, Jan Romantowski, Hanna Kujawska‐Danecka, Marek Niedoszytko

**Affiliations:** ^1^ Department of Allergology Medical University of Gdańsk Gdańsk Poland; ^2^ Department of Rheumatology Medical University of Gdańsk Gdańsk Poland

To the Editor,

Mastocytosis is a hematological neoplasm with a broad spectrum of disease caused by mast cell (MC) infiltration (i.e., impairment of function of bone marrow, liver) and degranulation (i.e., anaphylaxis).^1^ Recent data indicate psychological factors and neurological factors.^2^ Patients with mastocytosis have a significantly lower quality of life and experience increased anxiety, depression, and other symptoms such as headache or fatigue.^3^, ^4^ These might be accompanied by cognitive impairment and brain fog declared by 86% of US patients, though its relation with mastocytosis and its progression hasn't been established to date.^5^ Some of these symptoms might result from abnormal MCs in central nervous system.^6^ In murine model, Esposito et al. showed that stress through corticotropin stimulates brain MCs to disrupt permeability of brain–blood barrier. This effect was absent in MC‐depleted rodents and after cromolyn administration. The aim of the study was to investigate cognitive dysfunctions in patients with mastocytosis and analyze its connection with disease characteristics in patients.

The group of 79 Polish patients suffering from mastocytosis was enrolled (59 women and 20 men; age 20–75) after signing written informed consent. Fifty‐four of them suffered from indolent systemic mastocytosis, 2 had diffuse cutaneous mastocytosis, 20 had maculopapulous cutaneous mastocytosis, 2 had smoldering systemic mastocytosis, and there was a single patient with bone marrow mastocytosis. Serum tryptase ranged from 3 to 190 ng/ml with mean value 47.6 ng/ml. The education level of the studied population was high. Forty patients reported high education, 26 middle school, 10 vocational education, and 3 elementary.

All of the patients were evaluated with Mini–Mental State Examination (MMSE) that assesses cognitive functions in 0–30 point scale.^7^ The cut‐off point that suggests dementia is below 24 for educated populations, including Poland. All patients had bone marrow biopsy performed. Mastocytosis treatment was evaluated with WHO Performance Status; the patients were also asked about particular typical MC activation symptoms.^8^ Statistical analysis was conducted with Statistica software using Spearman correlation test. Cut‐off point for the *p*‐value was 0.05.

The mean MMSE score was 27.8 (median 29). Six out of 79 patients (7.6%) scored below 24 points (see Figure 1). Other 16 patients (20%) scored 24–26 points, which suggests minimal cognitive impairment (MCI).^7^ According to performance status, 23 patients had no symptoms, 55 scored 1, and one patient scored 2. There was a positive correlation with education level (*R* = 0.45) and a negative one with age (*R* = −0.34), as expected. MMSE correlated negatively with the presence of 25% atypical spindle‐shaped MCs in bone marrow (minor systemic mastocytosis criterion) with (*R* = −0.5) and bone pain (*R* = −0.45). No correlation was found between MMSE and serum tryptase level, presence of KIT bone marrow mutation, CD2/CD25 expression, skin involvement or performance status. A weak correlation was observed for the presence of CD30 (*R* = −0.1) and symptoms: pruritus (*R* = −0.13), edema (*R* = −0.23). There were no differences in results between patients with cutaneous and indolent systemic mastocytosis subgroups.

**FIGURE 1:**
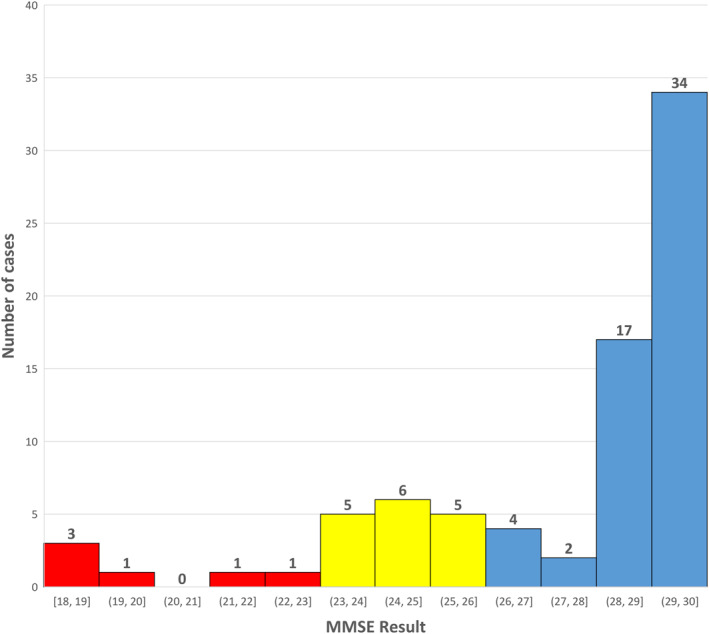
Number of cases with results of Mini‐Mental State Examination. Red color shows potential dementia; yellow color shows minimal cognitive impairment; and blue color shows normal results

The results of our study are in contrast to previously done surveys by Jennings et al.^5^ where 86% of the sample declared cognitive dysfunctions. However, our study suggests that such high cognitive impairment prevalence could have been over‐diagnosed. Using well‐known, validated MMSE method, we observed that only 7.6% of the sample experienced significant cognitive impairment while total 27% presented slight cognitive abnormalities. Such high self‐reported symptoms may be due to multiple emotional and personal problems, or affective disorders, that patients experience because of MC activation. Those might be misinterpreted by patients and labeled as cognitive dysfunctions.

The percentage of atypical MCs and bone pain that correlated to MMSE is associated with systemic mastocytosis and usually increases as the disease progresses to an aggressive form. Interestingly, there was no relation of MMSE to serum tryptase concentration, skin involvement, and general symptoms assessed by performance status. It is possible that cognitive dysfunctions might be linked to mastocytosis aggressiveness rather than to MC activation. In such case, according to Theoharides et al., serum IL‐6 might be a promising predictor of cognitive dysfunctions as it is related to MC‐related osteoporosis and bone pain.^9^ However, further studies are required in this area.

## KEYWORDS

education, mast cells, quality of life

## SCHLÜSSELWÖRTER

bildung, lebensqualita, mastozytose, mastzelle

## CONFLICT OF INTEREST

The authors declare no conflict of interests.

## FUNDING INFORMATION

Medical University of Gdansk, Grant/Award Number: ST 02‐141/07/231
